# Registered Nurses’ Experiences With Incivility During the Early Phase of COVID-19 Pandemic: Results of a Multi-State Survey

**DOI:** 10.1177/21650799211024867

**Published:** 2021-07-28

**Authors:** Mazen El Ghaziri, Susan Johnson, Christina Purpora, Shellie Simons, Rosemary Taylor

**Affiliations:** 1 University of Massachusetts Lowell; 2 University of Washington Tacoma; 3 University of San Francisco; 4 University of Massachusetts Medical School

**Keywords:** incivility, cyber-incivility, registered nurses, COVID-19, occupational stressors

## Abstract

**Background:**

Incivility among workers in the health sector is recognized as an occupational hazard. The COVID-19 outbreak brought sudden and profound changes to many health care settings, many of which have been identified as antecedents to workplace incivility. The purpose of this retrospective study was to explore the experiences of registered nurses with workplace incivility, cyber-incivility, and incivility outside of work during the early phase of the COVID-19 pandemic.

**Methods:**

This mixed-methods study used convenience sampling. Data were collected from June to September 2020 via an online survey, which consisted of both closed- and open-ended questions. Participants were recruited from national nursing organizations and unions. Data were analyzed using descriptive statistics and thematic analysis for open-ended responses.

**Findings:**

A total of 526 nurses’ responses were included in the analysis. More than one third experienced greater incivility at work during the COVID-19 outbreak than before the pandemic (37.4%), and almost half (45.7%) said they witnessed more incivility than before the pandemic. Cyber-incivility and incivility outside of work were also issues. Qualitative results indicated that respondents felt they were on edge during this period. Other themes included leadership failure, fractured co-worker relationships, heightened incivility from patients and families, and *hostility and ostracism from the general public*

**Conclusion/Application to practice:**

Occupational health nurses, nursing leaders, and staff nurses need to work to restore relations that were fractured by incivility during the pandemic. In the future, improved preparedness, including establishing clear channels of communication, may lessen incivility by decreasing role stress and organizational chaos.

Severe acute respiratory syndrome coronavirus 2 (SARS-CoV-2) first appeared in the United States in early 2020 and, by May 28 of that year, had resulted in 100,000 deaths (Centers for Disease Control and Prevention, 2020). The COVID-19 pandemic created new stressors and challenges for health care organizations and for the nurses who work in them, including rapidly changing guidelines on best practices for caring for COVID-19 patients (Markey et al., 2021). In addition, nurses were challenged with ongoing shortages of personal protective equipment (PPE; Cohen & Rodgers, 2020), and a surge of patients in intensive care units and emergency departments while other units such as outpatient surgery were shut down (Grimm, 2020). In this stressful environment, it is also likely that nurses were experiencing higher levels of incivility because workplace stressors, organizational change, increased job demands, and job insecurity have all been associated with workplace incivility (Torkelson et al., 2016). Role stress, defined as situations in which workers experience ambiguity, conflict, and overload, is an antecedent of workplace incivility (Schilpzand et al., 2016) that was present in health care settings during the COVID-19 pandemic (Markey et al., 2021).

Workplace incivility occurs when interpersonal interactions in the workplace are rude, discourteous, and in violation of norms for mutual respect (Schilpzand et al., 2016). In the nursing profession, high levels of incivility have previously been documented (Bambi et al., 2018). The American Nurses Association (2015) has called on organizations to adopt a zero-tolerance stance toward workplace incivility. Workplace incivility behaviors include interrupting, yelling, ignoring, and insulting other members of the workplace (Matthews & Ritter, 2015). Incivility is more than a nuisance. Incivility has been associated with poor mental health (Wing et al., 2015), lower levels of physical safety at work (Gazica & Spector, 2016; McGonagle et al., 2014), turnover intentions, and burnout (Rahim & Cosby, 2016). Incivility also has a direct effect on patient care, as health care providers who experience incivility are more likely to make mistakes that affect patient outcomes (Johnson et al., 2020; Riskin et al., 2015). Workplace incivility is like workplace bullying in its manifestation; however, unlike workplace bullying, it does not have to be sustained and can include single acts where there is no clear intention to create harm (Schilpzand et al., 2016).

During the COVID-19 pandemic, more clinical and non-clinical work was done virtually via online platforms (e.g., telemedicine and virtual staff meetings; Schwamm et al., 2020). This change in workplace operations had the potential for creating more opportunities for nurses to experience cyber-incivility, defined as “rude/discourteous behaviors occurring through Information and Communication Technologies (ICTs) such as e-mail or text messages” (Giumetti et al., 2012, p. 148). While cyber-incivility has been studied in the context of nursing education (Clark & Luparell, 2020), to our knowledge it has yet to be studied in the context of the clinical nursing workforce in the United States.

During the COVID-19 pandemic, there were media reports of health care workers experiencing incivility and aggression from the general public (White, 2020). While the World Health Organization (WHO, 2002a) includes violence against health care workers on their commute to and from work in their definition of workplace violence, this type of violence has not been fully investigated in the United States.

The purpose of this study was to explore nurses’ experiences with workplace incivility, cyber-incivility, and incivility outside of work during the early phase of the COVID-19 pandemic.

## Methods

This was a cross-sectional, descriptive study that used convenience sampling and collected data through an online questionnaire conducted from June to September 2020. The 26-item survey consisted of questions about incivility and cyber-incivility, workload, and worker demographics. Participants were registered nurses in the United States. Email announcements were sent separately to members of professional nursing organizations and unions in California, Massachusetts, New Hampshire, and Washington State. Collectively, they are the largest professional organizations and unions in the United States, representing over 112,000 registered nurses in these four states (Massachusetts Nurses Association, 2021; New Hampshire Nurses’ Association, 2020; Service Employees International Union, 2021; Washington State Nurses Association, 2021). Participants accessed the survey through a Qualtrics link provided in the email or social media posts of the professional nursing organizations and unions. The first page of the survey was a consent form. It was estimated that the survey would take 15 to 20 minutes to complete. Participation was voluntary and no incentives were given. Human subjects approval was provided by the University of Massachusetts Lowell, University of Washington Tacoma, and University of San Francisco.

### Data Collection

Incivility behavior was measured with an adapted version of the four-item Gender Invariant Measure of Workplace Incivility (Matthews & Ritter, 2016) with permission from the authors. The responses to each of the items used a 5-point Likert-type scale: *never* (0), *rarely* (1), *sometimes* (2), *frequently* (3), and *many times* (4). Using this scale, participants were asked to separately rate incivility perpetrated by co-workers and supervisors with these four items: paid little attention to my statements or showed little interest in my opinion, being interrupted or “spoken over,” being ignored or failed to be spoken to (e.g., gave you the silent treatment), and made jokes at your expense. A fifth item from the Negative Acts Questionnaire–Revised (Einarsen et al., 2009) was included to determine whether the participants were shouted at or whether they were made the target of spontaneous anger. This item was measured with the same 5-point Likert-type scale.

We used the definition for cyber-incivility developed by Giumetti et al. (2012) to create the following one-item measure of cyber-incivility: “During the COVID-19 pandemic I have experienced cyber-incivility from coworkers i.e. rude or discourteous behaviors occurring through phone conversations, email, text messaging, chat rooms, conferencing platforms, social media, or other remote means of communication.” The responses to this item used the same 5-point Likert-type scale. Following the above question, and to explore whether participants thought the amount of incivility during the COVID-19 pandemic was different compared with pre-pandemic, we asked participants whether they experienced incivility or cyber-incivility at work for the first time and whether it was the same as before, more than before, less than before, or not applicable (never experienced workplace incivility or cyber-incivility). This section also included the following open-ended question: “Describe a situation that best exemplifies the incivility or cyber-incivility that you have experienced during the COVID-19 pandemic.”

There were two items asking the respondent to identify the perpetrators of in-person and cyber-incivility with the following responses: registered nurses, supervisor/middle-level manager, patients, family and friends of patients, physicians, hospital administrator, unlicensed nursing staff (certified nursing assistant [CNA], unlicensed assistive personnel [UAP], other), non-nursing co-workers, licensed practical nurses/vocational nurses, others not listed (open ended). Multiple responses were allowable for the two perpetrator questions. In addition, two items assessed whether the participants witnessed incivility or acted in uncivil manner for the first time, the same as before, more than before, less than before, or not applicable (neither witnessed nor acted in uncivil manner).

Incivility on their commute to and from work was measured with a single-item yes/no question: “During the COVID-19 pandemic, I have experienced incivility related to my role as a health care professional on the way to or from work.” Participants who answered “yes” were asked to describe this experience.

To measure workload, we asked participants how many hours they were working, whether this was the same or different than before the pandemic, and whether they were still working in the same department or unit. Finally, the survey included demographic and work-related questions related to gender, age, racial identity, education level, occupational setting, position, years of employment as nurse, and the state in which the participant worked during the time covered by the survey.

### Data Analysis

SPSS (Version 25 for Mac, IBM Corp., Armonk, NY, USA) was used to calculate descriptive statistics, including frequency and percentage for categorical-level variables and mean and standard deviation for continuous variables. The five incivility behavior items were combined into two indices for incivility perpetrated by co-workers and by supervisors (Cronbach’s α of .86 for both) that were calculated by summing the individual items, where the scores ranged from 0 to 20, with a higher score represented more exposure. Missing data were excluded from the analyses for cases with no responses to all of the incivility behavior items by co-workers and supervisors using listwise deletion.

Responses to open-ended questions were analyzed by three of the researchers, one from each study region, using Braun et al.’s (2016) realist, semantic approach to thematic analysis. After reading their own data sets twice, each researcher coded their data and started to group the codes into candidate themes. Then, the three researchers met to discuss candidate themes. A preliminary framework of two overarching themes, four major themes, and several subthemes was developed. After separately comparing the data to the themes identified in this meeting, the three researchers met one more time to reach final agreement. To ensure the integrity of the analysis, the results of the qualitative analysis were then presented to the whole research team.

## Results

In total, 631 participants responded and 526 completed surveys were included in the analysis, primarily from Massachusetts (47.3%), Washington (32.2%), and California (11.7%) (Table 1). The sample was primarily female (93%) and White (87.3%), with a mean age of 45.8 years (±13.5 years). Most participants were staff nurses (89.3%) working in inpatient hospital settings (78%), and more than half had a bachelor’s degree (56.5%) with a mean job tenure of 18.7 years (±14.6 years). More than two thirds of the participants worked 25 to 40 hours per week (62.3%) and reported increased workload during the COVID-19 pandemic (67.4%) while less than half of the participants reported that they were reassigned to other units (41.3%). Most respondents were working in the same state as before the pandemic (99.5%).

**Table 1.** Demographic and Work-Related Characteristics of Registered Nurse Respondents, *N* = 526

Characteristic (*N* = 526)	Frequency (%) or *M* ± *SD*
Gender	
Female	402 (93)
Male	21 (4.8)
Gender queer	1 (0.2)
Chose not to answer	9 (2.0)
Age	45.8 ± 13.5 years
Racial identity	
White	315 (87.3)
African American	12 (3.3)
Asian	10 (2.8)
Hispanic	13 (3.6)
Mixed race or ethnicity	11 (3.0)
Education level	
Diploma and associate degree	101 (23.4)
Bachelor’s degree	244 (56.5)
Graduate degree (Master’s degree or higher)	87 (20.1)
State	
California	50 (11.7)
Massachusetts	203 (47.3)
Washington	138 (32.2)
Other states	28 (8.8)
Occupational setting	
Inpatient hospital	333 (78)
Outpatient clinic or primary care	28 (6.5)
Public/community health	26 (6.1)
Outpatient surgery	15 (3.5)
Long-term care	12 (2.8)
Currently not working	8 (1.5)
COVID-19 related	4 (0.9)
Mix of inpatient and outpatient	3 (0.7)
Position	
Staff nurse	383 (89.3)
Manager/administrator/director	26 (6.0)
Nurse educator	9 (2.1)
Different role	11 (2.6)
Years worked	18.7 ± 14.6 years
Workload during COVID-19 pandemic	
More than usual—Before COVID-19 pandemic	294 (67.4)
Same as usual—Before COVID-19 pandemic	90 (21.0)
Less than usual—Before COVID-19 pandemic	45 (10)
Furloughed or laid off	3 (0.7)
Not working due to health concerns	4 (0.9)
Hours worked per week	
1–24 hours	45 (10.0)
25–40 hours	271 (62.0)
More than 40 hours	112 (26.0)
I haven’t been working during the COVID-19 pandemic	7 (2.0)
Setting	
Reassigned to another unit	180 (41.3)
Same	235 (53.9)
Working from home	12 (2.8)
Not applicable (I am not working during COVID-19 pandemic)	9 (2.0)

*Note.* Numbers (*n*) on some variables may not sum to total due to missing data.

Greater than one third of the participants experienced more incivility during the COVID-19 pandemic than before (37.4%), and almost half (45.7%) said they witnessed more incivility than before the pandemic (Table 2). The perpetrators of incivility were mostly registered nurses (41.8%) and supervisors or middle-level managers (40.5%). This is also reflected in the finding that mean scores were higher for incivility perpetrated by co-workers (6.68 ± 4.37) than for incivility perpetrated by supervisors (5.33 ± 4.73). One third reported physicians, patients, and family and friends of patients as perpetrators of incivility. As respondents could pick more than one source of incivility, responses in this category add up to more than 100%.

**Table 2.** Registered Nurses’ Self-Reported Exposure to, and Perpetrators of, Incivility and Cyber-Incivility, *N* = 526

Variable (*N* = 526)	*n* (%) or *M* ± SD
Mean incivility score (co-workers)	6.68 ± 4.37
Mean incivility score (supervisors)	5.33 ± 4.73
Experiences of incivility at work	
First time during pandemic	15 (3.0)
Same as before	169 (34.2)
More than before	185 (37.4)
Less than before	73 (14.9)
Not applicable—Have never experienced workplace incivility	52 (10.5)
Perpetrators of incivility	
Registered nurses	220 (41.8)
Supervisor/middle-level manager	213 (40.5)
Patients	180 (34.2)
Family and friends of patients	168 (31.9)
Physicians	161 (30.6)
Hospital administrator	109 (20.7)
Unlicensed nursing staff (CNA, UAP, other)	77 (14.6)
Non-nursing co-workers	67 (12.7)
Licensed practical nurses/vocational nurses	9 (1.7)
Others not listed	25 (4.8)
Witnessed incivility at work	
First time during pandemic	7 (1.4)
Same as before	170 (34.4)
More than before	226 (45.8)
Less than before	56 (11.3)
Not applicable—Have never witnessed workplace incivility	35 (7.1)
Participant acted in uncivil manner	
First time during pandemic	35 (7.1)
Same as before	86 (17.4)
More than before	50 (10.1)
Less than before	56 (11.3)
Not applicable—Have never acted in uncivil manner	267 (54.1)
Experienced cyber-incivility	
Never	270 (56.8)
Rarely	82 (17.3)
Sometimes	87 (18.3)
Frequently	26 (5.5)
Many times	10 (2.1)
Experiences of cyber-incivility	
First time during pandemic	26 (5.5)
Same as before	61 (12.8)
More than before	125 (26.3)
Less than before	20 (4.2)
Not applicable—Have never experienced cyber-incivility	243 (51.2)
Perpetrators of cyber-incivility	
Registered nurses	121 (23.0)
Supervisor/middle-level manager	69 (13.1)
Family and friends of patients	44 (8.4)
Non-nursing co-workers	41 (7.8)
Hospital administrator	33 (6.3)
Physicians	32 (6.1)
Patients	22 (4.2)
Unlicensed nursing staff (CNA, UAP, other)	18 (3.4)
Licensed practical nurses/vocational nurses	6 (1.1)
Others not listed	28 (5.3)
Experienced incivility during the COVID-19 pandemic related to the role as health care professional on the way to and from work	
Yes	97 (21.8)
No	348 (78.2)

*Note.* Numbers (*n*) on some variables may not sum to total due to missing data. CNA = certified nursing assistant; UAP = unlicensed assistive personnel.

Forty-three percent of the participants experienced cyber-incivility, and 31.8% said it was either for the first time during the pandemic or more than before. Registered nurses (23%) were the primary perpetrators of cyber-incivility, followed by a supervisor or middle-level managers (13.1%). Twenty two percent of the participants reported experiencing incivility related to their role as health care professionals on their way to and from work. Few participants reported acting in an uncivil manner for the first time during the pandemic (7.1%); however, 10% said they were more uncivil than before the COVID-19 pandemic.

**Figure 1.**
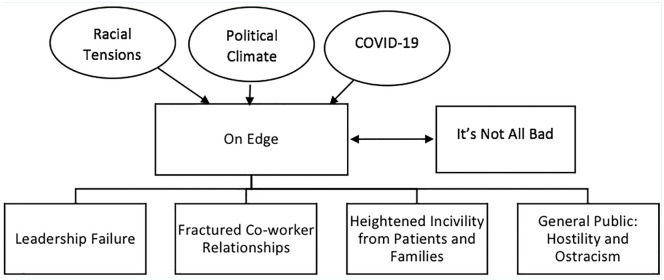
Quantitative themes. *Note.* The circles represent the societal stressors that respondents mentioned. The first row of rectangular shapes represents the two overarching themes. The second row represents the main themes within the overarching theme of “on edge.”

There were two overarching themes in the qualitative data, including on edge and it’s not all bad (Figure 1). Participants said people were “on edge,” or shorted tempered, irritable, and more stressed than usual. One wrote, “Staff and supervisor [*sic*] under more stress and patient workload is heavier. Tempers flare more quickly. Everyone seems more stressed in general.” Participants mentioned that they were not only on edge because of the pandemic but also because of the racial protests and political climate in the country. Despite the stressors they were facing, participants also stated that it’s not all bad and that incivility was not an issue before the pandemic in their workplace, and that it continued to be a non-issue. As one said, “I work at [HOSPITAL], it is a professional workplace and COVID has not changed that.”

The four major themes that were identified in the data were as follows: leadership failure, fractured co-worker relationships, heightened incivility from patients and families, and hostility and ostracism from the general public. These themes and their subthemes are discussed in the next section.

### Leadership Failure

Leadership failure was described as follows by the participants: Leadership did not address the rapidly changing crisis, they did not mitigate workplace incivility and other workplace stressors, and they perpetuated incivility. The theme of leadership failure is exemplified in the following: “What I have witnessed in these past few months due to the incompetence of this leadership team is beyond description.”

The subthemes in this category were as follows: absent, hostile, unreasonable demands and unsafe workplace conditions, downplaying, and cyber surveillance.

Leaders at all levels were described as being absent. Participants said, they ignored staff concerns about isolation protocols and equipment, ignored emails, they were not physically present on the unit (often working from home), or were sequestered in their offices behind closed doors. In this theme, participants described incivility as “Nursing voices not being heard by administration,” and “Manager and director of the unit not listening to our concerns, not sympathetic, and being ignored.”

As exemplified by the following quotes from different participants, nurses said managers met their requests for additional staff, information on policy updates, additional PPE, or other equipment with hostility and anger. “When questioning my manager about unilateral decisions made by admin that affect nurses at the bedside, I frequently get yelled at or told my concerns are invalid.” Another participant reported, “He [the manager] yelled at me in the hallway that the policy had changed and now everyone could wear the N95.”

As the pandemic progressed, nurses said that despite the increased acuity of COVID-19 patients, and the need to take extra precautions with all patients, staffing remained unchanged, use of overtime hours was questioned, and no accommodations were made for increased workload brought on by the pandemic. As in the following quote, they also described unsafe workplace conditions characterized by inadequate PPE and substandard patient care. In the words of one participant,

Administration just wanting you to take patients out of the ED with no thought to the census and staffing. [They told] us to go back to full census that would include [patients] sharing rooms/bathrooms/NO ability to distance. With no stable guidance/plan or safe regulations. Bare minimum PPE.

Nurses said their concerns about the virus, the need for proper PPE, patient screening, and isolation precautions were minimized by leadership. One described incivility as, “Toxic positivity! Downplaying people’s response to things saying, ‘they’re negative’ rather than really listening to the issues.” Incivility was also described as, “being told that our fears and concerns were not valid.”

Nurses said cyber-incivility from leadership manifested as monitoring of staffs’ workplace and personal social media and email accounts, which created an atmosphere of distrust. Cyber-incivility from leaders was described as, “a vague statement from upper management that came through our leadership that management was watching our personal social media posts.”

### Fractured Co-Worker Relationships

In this major theme, nurses said relations between co-workers were contentious and unsupportive, noting a lack of teamwork. Incivility which existed before the pandemic was described as ongoing. There were four subthemes: sitting in judgment of co-workers, frustration over perceived workplace inequities, lack of collegiality, and cyber-incivility.

Nurses described incidents where co-workers openly questioned their competence. New nurses, part-time nurses, and nurses who were not caring for COVID-19 patients felt they were singled out related to their lack of experience. One respondent said, there were “a few full-time RNs that are working in the COVID area of our unit [that] act like they are special or better than RNs that are per diem because we don’t work as much.” Another said they were “critiqued and criticized for not having expert skills,” but they were “working in unfamiliar territory, in a role not typical to normal role in very difficult times.”

Nurses described conflicts around patient assignments and workload. One said incivility manifested as,

nurses not wanting to accept a patient assignment who immediately point out what others are NOT doing stating “I just got one, you’re not being fair!” THEN spend the rest of the shift either avoiding you, muttering to themselves, or taking small digs at you.

Nurses who had underlying risk factors for COVID-19 complications said they were belittled for “not taking confirmed COVID positive patients.”

Nurses said there was an atmosphere of “very little collaboration” between different units, between nurses in different roles, and between professions (e.g., nursing and medicine), as in the following example: “Providers (MD, DO, PA, and NP) refused to come to the designated rooms and forced RNs to go beyond my comfortable scope of practice.” Lack of collegiality was also described as, “Competition among staff for hours. Hours reduced in my department due to low surgical volume.”

In addition to in-person incivility, participants said that cyber-incivility occurred between co-workers via organizational channels (e.g., email, texts, and other chat features), during online meetings, and via personal channels (e.g., Facebook and texts). Some of the cyber-incivility was described as a continuation of what occurred before the pandemic, as in the following:

A group of older nurses partake in a text group that they call “bitches and bullies.” . . . It was accidentally discovered when a nurse was showing a younger nurse something on her phone and a text came through and the group name displayed at the top of her phone. She tried to cover it up but it had already been seen. More information came out regarding this group of nurses and their “text circle” and what is said about some of the other nurses.

Other participants said that they experienced cyber-incivility due to the increased use of virtual spaces. For example, “Non-nursing co-workers are frequently rude or disrespectful during cyber meetings.”

### Heightened Incivility From Patients and Families

In this theme, nurses described that ongoing incivility from patients and families was intensified by the pandemic. This theme contained the subthemes of outrage in reaction to restrictions and cyber-incivility.

Nurses said that incivility from patients and families centered around restrictions due to COVID-19, including the need for increased screening, restrictions on visiting, and rules about masks. For example, “One patient, in front of 3 co-workers, looked me in the face and said ‘go to hell’ because I asked his wife to wear a mask.” Another wrote, “A boyfriend of a COVID patient snuck into the hospital and came to see his gf. When caught he refused to leave until we called security. He was angry with nurses and refused to wear a mask.”

Nurses said cyber-incivility included negative posts from patient’s family on social media platforms. One wrote, “Patient’s wife posted multiple videos to Facebook regarding her displeasure with the care provided to her husband. Took videos of nurses who came in the room and named names of nurses who she didn’t like.”

Within this theme, nurses said strangers yelled at them outside of work; family, friends, and acquaintances avoided them; and they experienced increased cyber-incivility on social media platforms. There were three subthemes: yelling, shunning, and cyber-incivility.

Nurses described being yelled at by strangers when they were in public places (e.g., public transportation, stores, and takeout restaurants) and were identified as health care workers. One wrote, “I have received hateful comments while I was wearing scrubs pumping gas and buying groceries.” Another wrote, “A customer in the grocery store overheard me speaking with another customer regarding my line of work and proceeded to berate and yell at me for being at the grocery store and ‘spreading the virus.’”

Nurses said they were refused service, and said neighbors “crossed the street to avoid them,” and that family and friends also avoided them. One wrote that they experienced

Hassling for being a nurse. Told that I am unsafe and unwelcome at certain places. For example, my family told me I was unwelcome to see my grandmother on her deathbed when the rest of the family all came to her home to see her off.

Another wrote, “I have been refused to get a meal and been told to leave a grocery store.”

Nurses described experiencing “pure hatred” and personal attacks from friends, family, and strangers on social media platforms. They also equated comments that COVID-19 is a hoax and disinformation about COVID-19 on social media with incivility. One wrote, “Nurses [are] targeted on social media as spreading disease, perpetuating a hoax, and lying about COVID-19 by friends and family, but also by internet trolls.” Another wrote, “People on social media who know what I do for a living, have told people to not come and see my home, which is on the market for sale!!”

## Discussion

The results of this study suggest that incivility and cyber-incivility are workplace hazards for nurses that have been exacerbated by the COVID-19 pandemic. Forty percent of respondents either reported experiencing incivility for the first time or more than before the pandemic, and almost half said they witnessed more incivility than before the pandemic. Thirty-four percent of respondents said the levels of incivility they experienced were unchanged by the pandemic. Taken together, the results indicate that a majority of the sample experienced incivility during the study period. Maunder (2004) reported that during the severe acute respiratory syndrome (SARS) outbreak in Toronto in 2003, health care workers said they felt increased levels of stress and experienced more conflict with co-workers. Likewise, health care workers in New Zealand said that after two major earthquakes (2010, 2011) in Queensland, they were exposed to increased aggression, bullying, and emotional outbursts from co-workers (Van Heugten, 2018).

The workload and working conditions of the sample were also changed by the pandemic. Sixty-seven percent of the sample reported working more hours than usual, and 41% said they were working in different units. As increased workload, workplace stressors, and role ambiguity have all been associated with workplace incivility by previous studies (Oyeleye et al., 2013; Roberts et al., 2011; Torkelson et al., 2016), it is not surprising that this sample reported increased levels of incivility during the study period. Other workplace stressors that respondents reported in the qualitative data included lack of collegiality between colleagues, being required to work in unsafe conditions, and incivility from patients and family.

Sixty percent of the sample indicated that supervisors, middle-level managers, or administrators were perpetrators of incivility. Qualitative analysis suggests that this incivility was perceived as a failure of leadership which manifested as lack of support, ignoring or downplaying concerns, or outright hostility. Nurses in this study reported they did not have the resources to protect themselves and there was no stable guidance or plan to ensure their safety. Health care workers in Toronto reported similar circumstances—ignoring requests for PPE, inadequate screening and isolating of patients, and ignoring or downplaying safety precautions—during the 2003 SARS outbreak (Maunder, 2004).

In addition to leadership failure, nurses described fractured relationships with co-workers. Mean incivility scores were higher for the category of co-workers, and almost 42% of the sample indicated that registered nurses were the source of the incivility they experienced. The qualitative data indicated that incivility between co-workers manifested as judgment, frustration over perceived work inequities, lack of collegiality, and cyber-incivility. Previous studies linked nurses’ relationships with co-workers with job satisfaction (Purpora & Blegen, 2015). In addition, when co-worker relations are supportive, nurses openly share information related to patient care; they ask each other questions, provide each other feedback and advice, and seek clarification or validation of information (Purpora & Blegen, 2015), all of which can affect patient outcomes.

In contrast to our findings, studies of nurse’s experiences during outbreaks of influenza, Middle East Respiratory Syndrome (MERS), and SARS reported high levels of professional camaraderie (Ives et al., 2009; Kim, 2018; Liu & Liehr, 2009). Nurses in these studies equated the experience with working together in battle (Ives et al., 2009; Kim, 2018; Liu & Liehr, 2009). Likewise, Tam et al. (2004) reported that during a SARS outbreak in Hong Kong, 69.7% of nurses said, “relationship with colleagues became closer and more supportive” (p. 1202). Some of our participants had the same experience, about 15% of our sample reported experiencing less incivility than before the pandemic, and the qualitative data included the theme of it’s not all bad.

Participants in our study also experienced heightened incivility from patients and their families. Incivility from patients and families toward nurses has been documented prior to the pandemic (Campana & Hammoud, 2015; Layne et al., 2019). However, qualitative analysis of the data from our study indicated that participants felt incivility from patients and families was different and more intense than before the pandemic. Some patients and families were uncivil to requests to wear masks and pushed back on visitation policies. It has been suggested that visitation policies need to be revisited (Siddiqi, 2020), an action that would remove one stressor from the nursing profession.

Our study found that nurses also experienced incivility outside of work. While we only asked about incivility on their way to and from work, in their responses to the open-ended question asking them to describe this incivility, participants described hostility and ostracism from their own family, friends, and the public. Dye et al. (2020) also reported that globally health care workers have experienced harassment, stigmatization, and bullying from the public related to COVID-19. Although health care providers have been stigmatized during previous pandemics (Maunder, 2004), it is possible that infodemics, defined as “an overabundance of information—some accurate and some not—that makes it hard for people to find trustworthy sources and reliable guidance when they need it” (WHO, 2020b), a common problem during the COVID-19 pandemic (Barua et al., 2020; Islam et al., 2020), contributed to this stigmatization. Infodemics can contribute to panic and mistrust (Smith et al., 2020), which may account for some of the incivility that nurses in this and other studies have reported.

COVID-19 illustrated how nurses can be both revered and maligned for the work they do (Amon, 2020; Bagcchi, 2020). The WHO’s (2003) definition of workplace violence includes situations where health care workers are “. . . abused, threatened or assaulted in circumstances related to their work, including commuting to and from work, involving an explicit or implicit challenge to their safety, well-being or health” (p. 2). There is a paucity of research on the topic of nurses’ experiences with incivility and violence outside of work in the United States. Our study indicates that it was an area of concern during the early phases of the COVID-19 outbreak, and it deserves more research and attention.

To our knowledge, this study is the first to document clinical nurses’ experiences with cyber-incivility in the United States. The issue has been studied in the academic context (Clark et al., 2012). Forty-four percent of the survey said they experienced cyber-incivility during the study period. While the majority of those who experienced it said it was “rare” or “sometimes,” almost 8% said they were exposed to cyber-incivility “frequently” or “many times.” While supervisors, managers, and administrators were the primary perpetrators of face-to-face incivility, registered nurse co-workers were the major perpetrators of cyber-incivility. The qualitative data indicated that cyber-incivility was also perpetrated by patients and their families. In addition, the sample indicated they were concerned by the general level of incivility they experienced in social media because of their role as nurses. Our evidence indicates that workplace cyber-incivility as well as the impact of cyber-incivility outside of work are work-related stressors that warrant attention and further research from occupational health professionals.

As the overarching theme from the qualitative data of on edge indicates, during the time this study was conducted, the United States was not only dealing with a pandemic but also with racial tensions, a contentious political climate, and a decline in civility in public discourse (Islam et al., 2020; Mello et al., 2020). Many of the participants wrote that the incivility they experienced was related to these issues, rather than COVID-19. While managers and occupational health nurses cannot control outside events, they can be aware that these events might affect workplace relationships and should remind staff of the need to always maintain professional and civil relationships.

### Implications for Occupational Health

The COVID-19 pandemic revealed deficiencies in epidemic preparedness which led to a lack of coordination to prevent and control the spread of the virus, and contributed to chaos and uncertainty within health care organizations (Islam et al., 2020). Preparedness and training for future disease outbreaks should be ongoing and must include all workers and the community (Smith et al., 2020). Better preparedness would have decreased the chaos, role ambiguity, and uncertainty that some of the nurses in our study wrote about, which in turn might have decreased the levels of incivility. In addition, educating the public about how infectious diseases spread, how the spread can be mitigated, and the need for short-term sacrifices (e.g., limitations on hospital visitation) during a disease outbreak might decrease some of the incivility nurses experienced from patients and their families, and outside of work. As a trusted profession, nurses who are involved in disaster planning should take a primary role in this outreach and education.

Occupational health workers should be aware that workplace incivility can occur outside of work hours, particularly during the commute. Nurses should be instructed to avoid wearing their scrubs or work badges outside of work. To facilitate this, organizations should provide nurses with uniforms that they can change into at work.

During the COVID-19 outbreak, nurses worldwide experienced immense pressure, fatigue, and stress (Mo et al., 2020). Occupational health workers need to be aware that incivility was an added stressor during this time. Research has identified an association between incivility and burnout (Oyeleye et al., 2013), and burnout has been linked to nurses’ intent to leave, decreased job satisfaction, and decreased patient satisfaction (Ferri et al., 2015; Kelly et al., 2021; Khamisa et al., 2016). Occupational health nurses and nursing leaders will need to find ways to support physically and emotionally exhausted workforce to mitigate some of the negative effects of burnout caused by this pandemic (Markey et al., 2021; Restauri & Sheridan, 2020).

This study represents a small sample of nurses, at a given period of time, which limits its generalizability. However, the quantitative and qualitative data from the different states were remarkably similar, which indicates that workplace incivility during the early phase of the COVID-19 outbreak was not confined to one region. Although findings cannot be generalized, the study revealed information about nurses’ experiences of incivility during this pandemic that may be useful to consider for planning for future infectious disease outbreaks. This study adds to the current literature to further document that co-worker incivility is an ongoing concern for nursing, and that cyber-incivility and incivility outside of work related to nurses’ professional roles are urgent issues that require prompt attention.

**Applying Research to Occupational Health Practice**

This study examined registered nurses’ experiences with incivility, cyber-incivility, and incivility outside of work during the early part of the COVID-19 pandemic in the United States. Respondents reported that they experienced increased levels of all three types of incivility during this time period. Incivility is an occupational stressor that has been associated with poor mental and physical health outcomes. Occupational health nurses need to consider this occupational hazard in their assessment of registered nurses, and need to be prepared to refer them to qualified practitioners if needed. When planning for future outbreaks, pandemics, or emergency situations, occupational health nurses need to be aware that incivility may spike and should be proactive in creating strategies to prevent it. Planning should include clarifying roles in advance of a pandemic, setting up clear communication channels, and providing education about effects of a pandemic on patients, families, and the general public.
